# Prime factorization algorithm based on parameter optimization of Ising model

**DOI:** 10.1038/s41598-020-62802-5

**Published:** 2020-04-28

**Authors:** Baonan Wang, Feng Hu, Haonan Yao, Chao Wang

**Affiliations:** 1 Key laboratory of Specialty Fiber Optics and Optical Access Networks, Joint International Research Laboratory of Specialty Fiber Optics and Advanced Communication, Shanghai Institute for Advanced Communication and Data Science, Shanghai University, Shanghai, 200444 China; 2 State Key Laboratory of Cryptology, P. O. Box 5159, Beijing, 100878 China; 3 Center for Quantum Computing, Peng Cheng Laboratory, Shenzhen, 518000 China

**Keywords:** Quantum physics, Quantum information

## Abstract

This paper provides a new (second) way, which is completely different from Shor’s algorithm, to show the optimistic potential of a D-Wave quantum computer for deciphering RSA and successfully factoring all integers within 10000. Our method significantly reduced the local field coefficient $h$ and coupling term coefficient $J$ by more than 33% and 26%, respectively, of those of Ising model, which can further improve the stability of qubit chains and improve the upper bound of integer factorization. In addition, our results obtained the best index (20-bit integer (1028171)) of quantum computing for deciphering RSA via the quantum computing software environment provided by D-Wave. Furthermore, Shor’s algorithm requires approximately 40 qubits to factor the integer 1028171, which is far beyond the capacity of universal quantum computers. Thus, post quantum cryptography should further consider the potential of the D-Wave quantum computer for deciphering the RSA cryptosystem in future.

## Introduction

The majority of scholars think that Shor’s algorithm is a unique and powerful quantum algorithm for the cryptanalysis of RSA. Therefore, the current state of the post quantum cryptography (constructing post quantum public key cryptosystems that would be secure against quantum computers) research has exclusively studied the potential threats to Shor’s algorithm.

The security of the RSA cryptography system is based on the high complexity and security of the integer factorization problem. Shor’s algorithm^1^ can attack the RSA cryptosystem in polynomial time. There have been many simulations about quantum computers^2^ and attempts to implement Shor’s algorithm on quantum computing hardware^3–7^. Researchers have developed classic emulators based on reconfigurable technology, enabling efficient simulation of various quantum algorithms and circuits, and they have the potential to simulate number of quits than software based simulators^2^. Nuclear Magnetic Resonance (NMR) is the technology that we have for the implementation of small quantum computers. Vandersypen *et al*.^8^ and Lu *et al*.^9^ applied Shor’s algorithm to factor the integer 15 via NMR and an optical quantum computer, respectively. Enrique *et al*. implemented a scalable version of Shor’s algorithm via the iterative approach to factor 21^10^. Based on the characteristics of the Fermat number^11^, Geller *et al*. used 8 qubits to successfully factor 51 and 85.

The real physical realizations of Shor’s algorithm cannot breakthrough the scale of factorization beyond 100 for the moment, as shown by principle-of-proof simulations and experiments^12^. Actually, the number of qubits for performing Shor’s algorithm to factor an n-bit integer still remains approximately 2n qubits^13^. Shor’s algorithm requires not only a large number of qubits but also a general-purpose quantum computer with high precision. Achieving practical quantum applications will take longer, perhaps much longer, as said by John Martinis, the physicist who leads Google’s efforts^14^, and Science^15^ commented that it will be years before code-cracking is achieved. Matthias Troyer said that “code-cracking and searching databases, are not good enough”^16^. The newest report by the National Academies of Sciences, “Quantum Computing: Progress and Prospects”, stated that the current state of quantum computing and progress is highly unlikely to be able to attack RSA 2048 within the next decade. Therefore, in the case where Shor’s algorithm cannot be practically applied, it is of great importance to find a more generalized and scalable way with the potential for practical attacks on integers while using fewer quantum resources.

The quantum adiabatic theorem was first introduced in 2001 by Burges^17^. The main idea is to construct the corresponding Hamiltonian based on the multiplication table^18–20^. Xu, N. *et al*. realized an experimental realization of factoring 143 via an NMR quantum processor^18^. By further employing the properties of some class of large integers, Dattani *et al*. factored the integer 56153 with only 4 qubits^19^ and Li *et al*. factored 291311 with 3 qubits by combining the theoretical reductions and Hamiltonian transformation^20^. However, these methods are only available for integers with special properties and cannot be generalized to large integers, which can merely be seen as a principle-of-proof experiment. In adiabatic quantum computation, some researchers^21,22^ realize the reduction of multiple terms to quadratic terms without introducing auxiliary qubits, but too many restrictions increase the complexity of the model. Thus, it is of great importance to find a more generalized way to conduct prime factorization.

D-Wave quantum computer is based on the quantum annealing principle. It has been widely used in sampling, optimization, machine learning, etc.^23–29^. Raouf Dridi *et al*.^27^ applied the computational algebraic geometry to transform the factorization problem to the QUBO model to be solved by the cell algorithm and the column algorithm respectively. The experiments via the D-Wave 2X show that dividing the columns to construct the Hamiltonian that is to be solved via quantum annealing can factor the integer 200099. Jiang *et al*.^30^ constructed a general model to factor the integer 376289 with 94 logical qubits via a D-Wave 2000Q System. However, it is still limited by the hardware restrictions of the quantum machine^31^. Peng *et al*.^32^ further promoted Jiang *et al.’s* work by reducing the number of qubits according to the constraints of the target values and the number of carrying numbers involved in the multiplication table. XinMei Wang^33^ commented that Peng *et al*.^32^ supported the optimistic potential of a D-Wave quantum computer for deciphering the RSA cryptosystem in the future. In 2019, Lockheed Martin’s Warren, R.H.^34^ proposed a chain factorization algorithm to factor all integers within 1000 by setting the upper limit of the factorability. However, this model uses more logical qubits, which means there is qubit redundancy.

In this work, we put forward a new independent model for prime factorization with few qubits to be solved by QA, and it successfully factors 1028171 via 88 qubits with the *qbsolv* software environment (the quantum computing software environment provided by D-Wave). This is superior to the results obtained by any other quantum algorithm, including Shor’s algorithm (factor up to 85) via different platforms (like the Hua-Wei quantum computing platform), quantum adiabatic computation via NMR (291311), and quantum annealing via the D-Wave platform (376289). Compared with ref. ^30^, in this paper, the local field coefficient $h$ and coupling term coefficient $J$ of Ising model are optimized to reduce the range of the model parameters, which reduces the coupling strength between qubits, further improves the stability of qubit chains and further improves the upper bound of the integer factorization. Our method has obtained the best index (20-bit integers (1028171)) of quantum computing for deciphering RSA, and it also exceeded the theoretical maximum (10-bit integers) of the IBM Q System One${}^{TM}$ with Shor’s algorithm, the work of Shuxian Jiang *et al*. (376289), and the maximum scale (7781) of Lockheed Martin’s Warren, R.H. It supports the optimistic potential of the quantum annealing algorithm and D-Wave quantum computer for deciphering the RSA cryptosystem in the future. The D-Wave provides a new (second) way, which is a completely different way than Shor’s algorithm, and may be closer to cracking practical RSA codes than a general-purpose quantum computer using Shor’s algorithm.

The rest of this paper is organized as follows. First, we describe the basic ideas of quantum annealing and the multiplication table for factorization. Second, we compare the methods and results with those of Shor’s algorithm, NMR, and integer factorization by a D-Wave. Third, we illustrate the optimistic potential of the quantum annealing algorithm and D-Wave quantum computer for deciphering the RSA cryptosystem. Finally, we point out that post quantum cryptography should not only consider the potential attacks from universal quantum algorithms, such as Shor’s algorithm but also consider real attacks from a D-Wave quantum computer in the near future.

## Methods

### Quantum annealing

Quantum annealing, as the core algorithm of a D-Wave quantum computer, has the potential to approach or even achieve the global optima in an exponential solution space, corresponding to the quantum evolution towards the ground state of the Hamiltonian problem^24^. The quantum processing units (QPUs), which are the core components for performing quantum annealing, are designed to solve quadratic unconstrained binary optimization (QUBO) problems^25,26^, where each qubit represents a variable, and the couplers between qubits represent the costs associated with qubit pairs.

The objective form of the QUBO that the QPU is designed to minimize is as follows:

1 $$Obj(x,Q)={x}^{T}\cdot Q\cdot x,$$

where $Obj$ represents objective function of QUBO, $x$ is a vector of binary variables of size $N$, and Q is an $N\times N$ real-valued matrix characterizing the relationship between the variables. Thus, any problem given in such a form can be solved by the D-Wave quantum annealer.

### Multiplication table for factorization

Quantum annealing uses the quantum effects generated by quantum fluctuations to realize the global optimal solution of the objective function. The integer factorization problem can be transformed into a combination optimization problem that can be handled by the quantum annealing algorithm, and the minimum energy value can be output through the quantum annealing algorithm. At this time, the minimum value is the successful solution of integer factorization. To clarify the integer factorization method via quantum annealing, we introduce a multiplication table to illustrate the feasibility of mapping the integer factorization problem to Ising model (a model can be processed by a D-Wave quantum computer). We illustrate the factorization of the integer multiplication table by factoring $N=p\times q$, where $p$ and $q$ are prime numbers. Table 1 shows the factorization of $143=11\times 13$. In Table 1, ${p}_{i}$ and ${q}_{i}$ represent the bits of the multipliers, and ${z}_{ij}$ is the carried bits from $i$th bit to the $j$th bit. All the variables ${p}_{i}$, ${q}_{i}$, and ${z}_{ij}$ in the equations are binary.

**Table 1:** Multiplication table for 143 = 11 × 13 in binary.

	2^7^	2^6^	2^5^	2^4^	2^3^	2^2^	2^1^	2^0^
p					1	${p}_{2}$	${p}_{1}$	1
q					1	${q}_{2}$	${q}_{1}$	1
					1	${p}_{2}$	${p}_{1}$	1
				${q}_{1}$	${p}_{2}{q}_{1}$	${p}_{1}{q}_{1}$	${q}_{1}$	
			${q}_{2}$	${p}_{2}{q}_{2}$	${p}_{1}{q}_{2}$	${q}_{2}$		
		1	${p}_{2}$	${p}_{1}$	1			
carries	${z}_{67}$	${z}_{56}$	${z}_{45}$	${z}_{34}$	${z}_{23}$	${z}_{12}$		
	${z}_{57}$	${z}_{46}$	${z}_{35}$	${z}_{24}$				
p × q = 143	1	0	0	0	1	1	1	1

Note: All of the variables involved in Table 1 can only take the values of $\left\{0,1\right\}$. Adding each column leads to the following equations:

2 $${p}_{1}+{q}_{1}=1+2{z}_{12}$$

3 $${p}_{2}+{p}_{1}{q}_{1}+{q}_{2}+{z}_{12}=1+2{z}_{23}+4{z}_{24}$$

4 $$1+{p}_{2}{q}_{1}+{p}_{1}{q}_{2}+1+{z}_{23}=1+2{z}_{34}+4{z}_{35}$$

5 $${q}_{1}+{p}_{2}{q}_{2}+{p}_{1}+{z}_{34}+{z}_{24}=0+2{z}_{45}+4{z}_{46}$$

6 $${q}_{2}+{p}_{2}+{z}_{45}+{z}_{35}=0+2{z}_{56}+4{z}_{57}$$

7 $$1+{z}_{56}+{z}_{46}=0+2{z}_{67}$$

8 $${z}_{67}+{z}_{57}=1.$$

Because each of the variables should be 0 or 1, we can get ${z}_{12}=0$ and ${p}_{1}{q}_{1}=0$ according to the equation ${p}_{1}+{q}_{1}=1+2{z}_{12}$. By applying similar judgments, we can get a simplified set of equations, as follows:

9 $${p}_{1}+{q}_{1}-1=0$$

10 $${p}_{2}+{q}_{2}-1=0$$

11 $${p}_{2}{q}_{1}+{p}_{1}{q}_{2}-1=0.$$

Obviously, ${({p}_{1}+{q}_{1}-1)}^{2}$, ${({p}_{2}+{q}_{2}-1)}^{2}$, and ${({p}_{2}{q}_{1}+{p}_{1}{q}_{2}-1)}^{2}$. The objective function is defined as the sum of squares of the three equations. It can be given as follows:

12 $$f={({p}_{1}+{q}_{1}-1)}^{2}+{({p}_{2}+{q}_{2}-1)}^{2}+{({p}_{2}{q}_{1}+{p}_{1}{q}_{2}-1)}^{2}.$$

It can be seen from the above that the minimum value of Eq. (12) is 0, that is, $$({p}_{1},{p}_{2},{q}_{1}$$, and $${q}_{2})$$ are the values that minimize Eq. (12), and it is also the solution of Eqs. (9)–(11). This means that the values of $$({p}_{1},{p}_{2},{q}_{1}$$, and $${q}_{2})$$ represent the solution to the factorization problem.

### The improved multiplication table for factorization

In the improved multiplication table for 143, ${c}_{1}$, ${c}_{2}$, ${c}_{3}$ and ${c}_{4}$ are the carried bits from the previous column. All the variables have a value of 0 or 1. Shuxian Jiang *et al*.^30^ divided the multiplication table into 4 columns (from right to left are column 1, column 2, column 3, and column 4), as shown in Table 2.

**Table 2:** Improved multiplication table for $143=11\times 13$ in binary.

	2^7^	2^6^	2^5^	2^4^	2^3^	2^2^	2^1^	2^0^
$p$					1	${p}_{2}$	${p}_{1}$	1
$q$					1	${q}_{2}$	${q}_{1}$	1
					1	${p}_{2}$	${p}_{1}$	
				${q}_{1}$	${p}_{2}{q}_{1}$	${p}_{1}{q}_{1}$	${q}_{1}$	
			${q}_{2}$	${p}_{2}{q}_{2}$	${p}_{1}{q}_{2}$	${q}_{2}$		
		1	${p}_{2}$	${p}_{1}$	1			
carries		${c}_{4}$	${c}_{3}$	${c}_{2}$	${c}_{1}$			
$p\times q=143$	1	0	0	0	1	1	1	1
		column 4		column 3		column 2		column 1

The equation for each column is as follows:

13 $$({p}_{2}+{p}_{1}{q}_{1}+{q}_{2}-({c}_{2}\times 4+{c}_{1}\times 2))\times 2+({p}_{1}+{q}_{1})={(11)}_{2}=3$$

14 $$({q}_{1}+{p}_{2}{q}_{2}+{p}_{1}+{c}_{2}-({c}_{4}\times 4+{c}_{3}\times 2))\times 2+(1+{p}_{2}{q}_{1}+{p}_{1}{q}_{2}+1+{c}_{1})={(01)}_{2}=1$$

15 $$(1+{c}_{4})\times 2+({q}_{2}+{p}_{2}+{c}_{3})={(100)}_{2}=4$$

Equations (13)–(15) are further simplified to the following

16 $$2{p}_{2}+2{p}_{1}{q}_{1}+2{q}_{2}-8{c}_{2}-4{c}_{1}+{p}_{1}+{q}_{1}-3=0$$

17 $$2{q}_{1}+2{p}_{2}{q}_{2}+2{p}_{1}+2{c}_{2}-8{c}_{4}-4{c}_{3}+{p}_{2}{q}_{1}+{p}_{1}{q}_{2}+{c}_{1}+1=0$$

18 $${q}_{2}+{p}_{2}+{c}_{3}+2{c}_{4}-2=0$$

We define the objective function as the sum of the squares of all the columns as follows:

19 $$\begin{array}{lll}f & = & {(2{p}_{2}+2{p}_{1}{q}_{1}+2{q}_{2}-8{c}_{2}-4{c}_{1}+{p}_{1}+{q}_{1}-3)}^{2}\\  &  & +\,{(2{q}_{1}+2{p}_{2}{q}_{2}+2{p}_{1}+2{c}_{2}-8{c}_{4}-4{c}_{3}+{p}_{2}{q}_{1}+{p}_{1}{q}_{2}+{c}_{1}+1)}^{2}\\  &  & +\,{({q}_{2}+{p}_{2}+{c}_{3}+2{c}_{4}-2)}^{2}.\end{array}$$

Since Ising model can only deal with the interaction of two variables, it is necessary to process polynomials greater than the 2-local term. According to the properties ${p}^{2}=p$, ${q}^{2}=q$, and ${c}^{2}=c$ (the values of $p$, $q$ and $c$ are 0 or 1), Eq. (19) is expanded and simplified, and the polynomials of more than 2-local term are replaced by the following equation^30^ (for more information about factorization refer to ref. ^30^):

20 $$\left\{\begin{array}{l}{x}_{1}{x}_{2}{x}_{3}=\mathop{\min }\limits_{{x}_{4}}({x}_{4}{x}_{3}+2({x}_{1}{x}_{2}-2{x}_{1}{x}_{4}-2{x}_{2}{x}_{4}+3{x}_{4}))\\ -{x}_{1}{x}_{2}{x}_{3}=-\mathop{\min }\limits_{{x}_{4}}({x}_{4}{x}_{3}+2({x}_{1}{x}_{2}-2{x}_{1}{x}_{4}-2{x}_{2}{x}_{4}+3{x}_{4})).\\ \end{array}\right.$$

We replace ${p}_{1}{q}_{1}$, ${p}_{1}{q}_{2}$, ${p}_{2}{q}_{2}$, and ${p}_{2}{q}_{1}$ with ${t}_{1}$, ${t}_{2}$, ${t}_{3}$, and ${t}_{4}$, respectively. In Eq. (20), the variable ${x}_{i}$ is used to represent the rule that the cubic term is reduced to the 2-local term. For example, the expansion term ${p}_{1}{q}_{1}{q}_{2}$ in Eq. (19) is replaced by ${t}_{1}{q}_{2}+2({p}_{1}{q}_{1}-2{p}_{1}{t}_{1}-2{q}_{1}{t}_{1}+3{t}_{1})$. Then, we perform variable replacement to transform the variables into the domain $0,1$ by using $${x}_{i}=(1-{s}_{i})/2,i=1,2,3,\cdots \ $$ if we let ${x}_{1}={p}_{1}$, ${x}_{2}={p}_{2}$, ${x}_{3}={q}_{1}$, ${x}_{4}={q}_{2}$, ${x}_{5}={c}_{1}$, ${x}_{6}={c}_{2}$, ${x}_{7}={c}_{3}$, ${x}_{8}={c}_{4}$, ${x}_{9}={t}_{1}$, ${x}_{10}={t}_{2}$, ${x}_{11}={t}_{3}$, and ${x}_{12}={t}_{4}$. Finally, via the correspondence ${p}_{1}={s}_{1}$, ${p}_{2}={s}_{2}$, ${q}_{1}={s}_{3}$, $${q}_{2}={s}_{4}\cdots \ $$, ${t}_{3}={s}_{11}$, and ${t}_{4}={s}_{12}$, Eq. (19) finally simplifies to the following:

21 $$\begin{array}{lll}f{\prime}  & = & ({p}_{1},{p}_{2},{q}_{1},{q}_{2},{c}_{1},{c}_{2},{c}_{3},{c}_{4},{t}_{1},{t}_{2},{t}_{3},{t}_{4})\\  & = & (261{s}_{1})/2+(215{s}_{2})/2+(261{s}_{3})/2+(215{s}_{4})/5-41{s}_{5}-82{s}_{6}+3{s}_{7}+6{s}_{8}-137{s}_{9}-81{s}_{10}-107{s}_{11}-81{s}_{12}+2{s}_{1}{s}_{2}+79{s}_{1}{s}_{3}\\  &  & +\,(95{s}_{1}{s}_{4})/2+(95{s}_{2}{s}_{3})/2-2{s}_{1}{s}_{5}+71{s}_{2}{s}_{4}-4{s}_{1}{s}_{6}-8{s}_{2}{s}_{5}+2{s}_{3}{s}_{4}-8{s}_{1}{s}_{7}-16{s}_{2}{s}_{6}-2{s}_{3}{s}_{5}-16{s}_{1}{s}_{8}\\  &  & +\,{s}_{2}{s}_{7}-4{s}_{3}{s}_{6}-8{s}_{4}{s}_{5}-148{s}_{1}{s}_{9}+2{s}_{2}{s}_{8}-8{s}_{3}{s}_{7}-16{s}_{4}{s}_{6}-84{s}_{1}{s}_{10}+6{s}_{2}{s}_{9}-16{s}_{3}{s}_{8}+{s}_{4}{s}_{7}+34{s}_{5}{s}_{6}\\  &  & +\,6{s}_{2}{s}_{10}-148{s}_{3}{s}_{9}+2{s}_{4}{s}_{8}-4{s}_{5}{s}_{7}-124{s}_{2}{s}_{11}+6{s}_{4}{s}_{9}-8{s}_{5}{s}_{8}-8{s}_{6}{s}_{7}-84{s}_{2}{s}_{12}-84{s}_{4}{s}_{10}-8{s}_{5}{s}_{9}-16{s}_{6}{s}_{8}\\  &  & -\,84{s}_{3}{s}_{12}-124{s}_{4}{s}_{11}+{s}_{5}{s}_{10}-16{s}_{6}{s}_{9}+34{s}_{7}{s}_{8}+6{s}_{4}{s}_{12}+2{s}_{5}{s}_{11}+2{s}_{6}{s}_{10}+{s}_{5}{s}_{12}+4{s}_{6}{s}_{11}\\  &  & -\,4{s}_{7}{s}_{10}+2{s}_{6}{s}_{12}-8{s}_{7}{s}_{11}-8{s}_{8}{s}_{10}-4{s}_{7}{s}_{12}-16{s}_{8}{s}_{11}-8{s}_{8}{s}_{12}+{s}_{9}{s}_{11}+794\end{array}$$

The local field $h$ represents the coefficient value of the single term of all ${s}_{i}$ variables, and the coupling $J$ is the coefficient value of the 2-local term for all ${s}_{i}{s}_{j}$ variables. The final model can be given as follows:

22 $$h=[\begin{array}{cccccccccccc}130.5 & 107.5 & 130.5 & 107.5 & -41 & -82 & 3 & 6 & -137 & -81 & -107 & -81\\ \end{array}]$$

23 $$J=\left[\begin{array}{cccccccccccc} & 2 & 79 & 47.5 & -2 & -4 & -8 & -16 & -148 & -84 & 0 & 0\\  &  & 47.5 & 71 & -8 & -16 & 1 & 2 & 6 & 6 & -124 & -84\\  &  &  & 2 & -2 & -4 & -8 & -16 & -148 & 0 & 0 & -84\\  &  &  &  & -8 & -16 & 1 & 2 & 6 & -84 & -124 & 6\\  &  &  &  &  & 34 & -4 & -8 & -8 & 1 & 2 & 1\\  &  &  &  &  &  & -8 & -16 & -16 & 2 & 4 & 2\\  &  &  &  &  &  &  & 34 & 0 & -4 & -8 & -4\\  &  &  &  &  &  &  &  & 0 & -8 & -16 & -8\\  &  &  &  &  &  &  &  &  & 0 & 1 & 0\\  &  &  &  &  &  &  &  &  &  & 0 & 0\\  &  &  &  &  &  &  &  &  &  &  & 0\\ \end{array}\right]$$

Then, the model given in Eqs. (22)-(23) can be directly solved by the D-Wave machine or the *qbsolv* software environment can be used to perform the quantum annealing algorithm. In this way, the model for the factorization can be generalized to any integer. Furthermore, it is a scalable model for any large integer in theory and it is a real potential application for D-Wave.

In the case when the factorization increases in Shuxian Jiang *et al*.^30^, the growing number of qubits and the huge coupler strength in the theoretical quantum model will result in a nontrivial impact on the QA precision in the real D-Wave machine. Especially for limit-connectivity hardware, too high of costs regarding the number of qubits greatly limits the generalization and scalability of the factorization in large cases. In addition, the reduction from the 3-local term to the 2-local term increases the coupler strength and local field coefficient, especially for large integers.

This paper proposes a new model that addresses two perspectives: saving qubit resources and simplifying the quantum model to factor larger integers with fewer qubits. Using this way, we can reduce the number of involved qubits and the range of the coupler strength between qubits without any loss of generalization. It is expected to solve larger integers with fewer qubits so that the D-Wave can provide a more powerful capacity to factor large integers in the future.

### Optimization of model parameters

In Ising model in ref. ^30^, they did not consider the restrictions on the final model derived from the target values, which may cause too many carries to be involved in the model. Here we introduce the constraints derived from the difference between the target values and the maximal output of each column. The carries involved can be directly removed in some cases.

As shown in the improved multiplication table of Table 2, because all variables have values of $0,1$, according to the first entry ${p}_{1}+{q}_{1}=1$ of column 2, ${p}_{1}{q}_{1}=0$ can be obtained. The second entry ${p}_{2}+{p}_{1}{q}_{1}+{q}_{2}=1$ in column 2 is simplified to ${p}_{2}+{q}_{2}=1$. Therefore, there is no carry from column 2 to column 3, that is, ${c}_{1}=0$ and ${c}_{2}=0$. Thus, only two carries (${c}_{3}$ and ${c}_{4}$) are needed to represent the carry from column 3 into column 4. In addition, we can get ${p}_{1}=1-{q}_{1}$ and ${p}_{2}=1-{q}_{2}$ according to ${p}_{1}+{q}_{1}=1$ and ${p}_{2}+{q}_{2}=1$, respectively. Finally, the factorization of 143 only requires 5 qubits, a significant improvement compared to the original model with 12 qubits^30^.

Based on the optimization of ref. ^32^, the final parameters of the model are as follows:

24 $$h=\left[\begin{array}{ccccc}-25 & -50 & 60 & 60 & -120\\ \end{array}\right]$$

25 $$J=\left[\begin{array}{ccccc} & 34 & -4 & -4 & 8\\  &  & -8 & -8 & 16\\  &  &  & 41 & -96\\  &  &  &  & -96\\ \end{array}\right]$$

Actually, the method of ref. ^32^ is designed to reduce the number of qubits, and thus the improvements to the complexity of the model are limited. The main reason is that there is a “2” in Eq. (20), which leads to many high coupler strengths and local field coefficients in the final Hamiltonian resulting in fragile quantum states. Therefore, another optimization should be proposed to solve the above problem without the loss of generalization and scalability.

As mentioned above, we mainly focus on the optimization of the model parameters. Jiang *et al*.^30^ a way to reduce the 3-local term to a 2-local term, which increased the local field coefficient and coupler strength parameters, especially for large integers. In the integer factorization problem based on quantum annealing, the reduction of the model parameters is beneficial to reducing the hardware requirements and the precision of quantum annealing. To reduce the 3-local term to a 2-local term in the integer factorization process, inspired by ref. ^35^, we optimize Eq. (20) of ref. ^32^ and form a new dimension reduction method from the 3-local term to 2-local term, as shown in Eq. (26)

26 $$\left\{\begin{array}{l}\left.{x}_{1}{x}_{2}{x}_{3}=\mathop{\min }\limits_{{x}_{4}}({x}_{4}{x}_{3}+{x}_{1}{x}_{2}-{x}_{1}{x}_{4}-{x}_{2}{x}_{4}+{x}_{4})\right)\\ -{x}_{1}{x}_{2}{x}_{3}=-\mathop{\min }\limits_{{x}_{4}}({x}_{4}{x}_{3}+2({x}_{1}{x}_{2}-2{x}_{1}{x}_{4}-2{x}_{2}{x}_{4}+3{x}_{4})).\end{array}\right.$$

The negative term $$-{x}_{1}{x}_{2}{x}_{3}=-\mathop{\min }\limits_{{x}_{4}}({x}_{4}{x}_{3}+2({x}_{1}{x}_{2}-2{x}_{1}{x}_{4}-2{x}_{2}{x}_{4}+3{x}_{4}))$$ is the same as ref. ^30^. We mainly prove our optimization of the positive term, that is, why the positive term $${x}_{1}{x}_{2}{x}_{3}=\mathop{\min }\limits_{{x}_{4}}({x}_{4}{x}_{3}+{x}_{1}{x}_{2}-{x}_{1}{x}_{4}$$ $$-{x}_{2}{x}_{4}+{x}_{4})$$ holds.

27 $$\begin{array}{l}{x}_{1}{x}_{2}{x}_{3}=\mathop{\min }\limits_{{x}_{4}}({x}_{4}{x}_{3}+{x}_{1}{x}_{2}-{x}_{1}{x}_{4}-{x}_{2}{x}_{4}+{x}_{4})\\ \end{array}$$

Table 3 is a combination of 16 values of ${x}_{1}$, ${x}_{2}$, ${x}_{3}$, and ${x}_{4}$. The values of ${x}_{1}$, ${x}_{2}$, ${x}_{3}$, and ${x}_{4}$ are 0 or 1. The output of is given in the last column, followed by $\surd$ or $\times$ to represent whether ${x}_{1}{x}_{2}{x}_{3}$ equals ${\min }_{{x}_{4}}({x}_{4}{x}_{3}+{x}_{1}{x}_{2}-{x}_{1}{x}_{4}-{x}_{2}{x}_{4}$ + *x*_4_) or not. As mentioned earlier, the integer factorization problem is the problem of finding the minimum value of a function. In other words, solving the minimum value of ${x}_{1}{x}_{2}{x}_{3}$ is the same as solving ${\min }_{{x}_{4}}({x}_{4}{x}_{3}+{x}_{1}{x}_{2}-{x}_{1}{x}_{4}-{x}_{2}{x}_{4}+{x}_{4})$). Take the first two rows of the Table 3 as an example for the following illustration.

**Table 3:** The truth table for the dimension reduction.

${{\boldsymbol{x}}}_{1}$	${{\boldsymbol{x}}}_{2}$	${{\boldsymbol{x}}}_{3}$	${{\boldsymbol{x}}}_{4}$	${{\boldsymbol{x}}}_{1}{{\boldsymbol{x}}}_{2}{{\boldsymbol{x}}}_{3}$	Whether Eq. (27) achieves the minimum
0	0	0	0	0	0/$\surd$
0	0	0	1	0	1/$\times$
0	0	1	0	0	0/$\surd$
0	0	1	1	0	2/$\times$
0	1	0	0	0	0/$\surd$
0	1	0	1	0	0/$\surd$
0	1	1	0	0	0/$\surd$
0	1	1	1	0	1/$\times$
1	0	0	0	0	0 /$\surd$
1	0	0	1	0	0/$\surd$
1	0	1	0	0	0/$\surd$
1	0	1	1	0	1/$\times$
1	1	0	0	0	1/$\times$
1	1	0	1	0	0/$\surd$
1	1	1	0	1	1/$\surd$
1	1	1	1	1	1/$\surd$

In this case, where $${x}_{1}=0$$, $${x}_{2}=0$$, and $${x}_{3}=0$$ are fixed, $${x}_{1}{x}_{2}{x}_{3}=0$$; when $${x}_{4}=0$$, $${x}_{4}{x}_{3}+{x}_{1}{x}_{2}-{x}_{1}{x}_{4}-{x}_{2}{x}_{4}$$ + *x*_4_ = 0; when $${x}_{4}=1$$, $${x}_{4}{x}_{3}+{x}_{1}{x}_{2}-{x}_{1}{x}_{4}-{x}_{2}{x}_{4}+{x}_{4}=1$$. Therefore, $$\mathop{\min }\limits_{{x}_{4}}({x}_{4}{x}_{3}+{x}_{1}{x}_{2}-{x}_{1}{x}_{4}-{x}_{2}{x}_{4}+{x}_{4})=0$$. At this time, $${x}_{1}{x}_{2}{x}_{3}$$ is equivalent to $${\min }_{{x}_{4}}({x}_{4}{x}_{3}+{x}_{1}{x}_{2}-{x}_{1}{x}_{4}-{x}_{2}{x}_{4}+{x}_{4})$$, and so $${x}_{1}{x}_{2}{x}_{3}={\min }_{{x}_{4}}$$$$({x}_{4}{x}_{3}+{x}_{1}{x}_{2}-{x}_{1}{x}_{4}$$ $$-{x}_{2}{x}_{4}+{x}_{4})$$.

The dimension reduction method in this paper is not only applicable to the integer 143, but it is also applicable to the case where the polynomial of the objective function of any integer is greater than the quadratic term, such as the factorization of the 20-bit integer 1028171. A detailed analysis of the factorization is shown in the supplemental material. The method is universal and extensible. We do the following analysis. Assume that the objective function of the integer factorization is as follows:

28 $$S{(x)}_{min}=g(x)+f({x}_{i},{x}_{j},{x}_{k}),$$

where $g(x)$ and $f({x}_{i},{x}_{j},{x}_{k})$ are polynomials composed of two-local terms and 3-local terms, respectively. Then, it can be transformed based on Eq. (27) as follows:

29 $$S{\prime} {(x)}_{\mathop{\min }\limits_{{x}_{n}}}=g(x)+\mathop{\min }\limits_{{x}_{n}}({x}_{n}{x}_{k}+{x}_{i}{x}_{j}-{x}_{i}{x}_{n}-{x}_{j}{x}_{n}+{x}_{n}).$$

Therefore, the minimum value that solves the objective function $S{(x)}_{min}$ is equivalent to the minimum value of solving the 3-local term $f(x)$, namely, the value of ${\min }_{{x}_{n}}({x}_{n}{x}_{k}+{x}_{i}{x}_{j}-{x}_{i}{x}_{n}-{x}_{j}{x}_{n}+{x}_{n})$. Therefore, the objective function $S{(x)}_{min}$ has the same solution as $S{\prime} {(x)}_{\mathop{\min }\limits_{{x}_{n}}}$. Similarly, we analyze the 4-local term in the function. $f({x}_{i},{x}_{j},{x}_{k},{x}_{l})$ is a polynomial composed of 4-local terms. We consider ${x}_{k}$ and ${x}_{l}$ as a whole, and obtain Eq. (30) via ${\min }_{{x}_{n}}({x}_{n}{x}_{k}+{x}_{i}{x}_{j}-{x}_{i}{x}_{n}-{x}_{j}{x}_{n}+{x}_{n})$.

30 $$f({x}_{i},{x}_{j},{x}_{k},{x}_{l})=\mathop{\min }\limits_{{x}_{n}}({x}_{n}{x}_{k}{x}_{l}+{x}_{i}{x}_{j}-{x}_{i}{x}_{n}-{x}_{j}{x}_{n}+{x}_{n}).$$

For the 3-local term ${x}_{n}{x}_{k}{x}_{l}$ in Eq. (30), the dimensionality reduction formula ${\min }_{{x}_{n}}({x}_{n}{x}_{k}+{x}_{i}{x}_{j}-{x}_{i}{x}_{n}-{x}_{j}{x}_{n}+{x}_{n})$ is used again to obtain the following:

31 $$f({x}_{n},{x}_{k},{x}_{l})=\mathop{\min }\limits_{{x}_{m}}({x}_{m}{x}_{l}+{x}_{n}{x}_{k}-{x}_{n}{x}_{m}-{x}_{k}{x}_{m}+{x}_{m}).$$

Finally, the final 4-local term is reduced to a 2-local term as follows:

32 $$f({x}_{i},{x}_{j},{x}_{k},{x}_{l})=\mathop{\min }\limits_{{x}_{n}}(\mathop{\min }\limits_{{x}_{m}}({x}_{m}{x}_{l}+{x}_{n}{x}_{k}-{x}_{n}{x}_{m}-{x}_{k}{x}_{m}+{x}_{m})+{x}_{i}{x}_{j}-{x}_{i}{x}_{n}-{x}_{j}{x}_{n}+{x}_{n}).$$

In this way, the minimum value of the 3-local term and 4-local term can be transformed to a simpler polynomial with simple connections characterized by quadratic terms. The coupler strength and local field coefficient can be reduced further and the theoretical model can work better to describe the original problem with high precision in the simulations.

### Simulations

All the simulations are performed via MATLAB 2014 and Python 3.6 with the *qbsolv* software environment (provided by D-Wave), which can successfully factor 1028171. For more information about the integer 1028171, please refer to the supplemental material. Table S1 of the supplemental material shows the factorization of integer 1028171. The *qbsolv* software environment is a decomposition solver that finds the minimum value given by a QUBO problem by splitting it into pieces that are solved either via a D-Wave system or a classical tabu solver. For more information about the tool, please refer to *http://github.com/dwavesystems/qbsolv*.

The simulations are based on the combination of the two optimizations, which can be divided into the following steps.

- Step 1. Give the improved multiplication table of Jiang *et al*.^30^ that is divided into several columns. It’s complexity is less than $O(lo{g}_{2}(N))$.
- Step 2. Give the original model based on the optimization in ref. ^32^. The complexity of this step is less than $O({(lo{g}_{2}(N))}^{3})$.
- Step 3. Give the final QUBO model based on the optimization of the model parameters. It’s complexity is less than $O({(lo{g}_{2}(N))}^{3})$.
- Step 4. Transform it to Ising model via $${x}_{i}=(1-{s}_{i})/2,i=1,2,3,\cdots \ $$, which is required for the quantum computing software environment. Note: ${x}_{i}$ variables are mapped to ${s}_{i}$ variables that could be processed by Ising model by the formula $${x}_{i}=(1-{s}_{i})/2,i=1,2,3,\cdots \ $$. The complexity of this step is $O(1)$.
- Step 5. Perform the simulations using the quantum computing software environment. By inputting the parameter values of $h$ and $J$ in the *qbsolv* quantum computing software, the quantum annealing algorithm factors the integers. It’s complexity is less than $O({(lo{g}_{2}(N))}^{2})$.

In the above simulations, Steps 1–4 are classical calculations, and the complexity is less than $O({(lo{g}_{2}(N))}^{3})$. Step 5 performs a quantum annealing calculation. The complexity increases as the integer to be factored becomes larger, and the overall complexity is less than $O({(lo{g}_{2}(N))}^{2})$. This algorithm realizes the hybrid computing structure of quantum and classical, and exerts the optimal computing power of the distributed processing problem of both quantum and classical.

Take the factorization on 143 as an example, the final input is given as follows:

33 $$h=[\begin{array}{ccccc}-12 & -50 & -25 & 12 & -24\\ \end{array}]$$

34 $$J=\left[\begin{array}{ccccc} & 34 & -4 & -4 & 8\\  &  & -8 & -8 & 16\\  &  &  & 17 & -24\\  &  &  &  & -24\\ \end{array}\right]$$

## Results

Due to the accuracy of the error correcting and quantum manipulation technique, the short-time decoherence, the susceptibility to various noises, etc., the progress of universal quantum devices is slow, which limits the development and practical applications of Shor’s algorithm. The maximum factorization ability of Shor’s algorithm is currently the integer 85. However, D-Wave quantum computers have rapidly developed, and the number of qubits has been doubling every other year. Based on the quantum annealing method, we factor the integer 1028171. Although our method requires more qubits than Shor’s algorithm to factor the same integer, Shor’s algorithm is highly dependent on high-precision hardware. Actually, Science, Nature, and the National Academies of Sciences (NAS) are consistent in that it will be years before code-cracking by a universal quantum computer is achieved.

The existing works based on NMR utilize the special properties of certain primes to perform principle-of-proof experiments. The maximum integer of factorization based on an NMR platform is 291311. The integer factorization method based on the NMR platform is not applicable to all integers and is not universal and scalable.

Actually, our method is general and can factor up to 20-bit (1028171) integers, making it superior to the results obtained by any other physical implementations, including general-purpose quantum platforms (the Hua-Wei quantum computing platform), and far beyond the theoretical value (factor up to 10-bit integers) that can be obtained by the latest IBM Q System One${}^{TM}$ if it can run Shor’s algorithm.

Table 4 shows the parameter values of Jiang *et al.’s* method^30^ for integer factorization (please note that all the data of ref. ^30^ are given via our simulations, just for reference). Table 5 shows the factorization results of our method for the integers 143, 59989, 376289, 1005973 and 1028171. It can be seen from Table 5 that our method can successfully factor the integers 1005973 and 1028171. Jiang *et al.’s* method can factor up to the integer 376289, whereas ours method can achieve the factorization of the integer 1028171, making it superior to the results obtained by any other physical implementations. The reduction of the qubits can reduce the hardware requirements of the quantum annealing machine and further boost the accuracy of quantum annealing, which has great practical significance. In the case of the hardware restrictions of the quantum machine, our goal is to achieve the factorization of a larger-scale integer 1028171 with fewer qubits, which is the best integer factorization result solved by the quantum algorithm.

**Table 4:** The parameter values of Jiang *et al*.’s^30^ method for integer factorization.

Integers	$p\times q$	h ranges	J ranges
143	$11\times 13$	[$-137$, 130.5]	[$-148$, 79]
59989	$251\times 239$	[$-1842$, 2947]	[$-1832$, 921]
376289	$659\times 571$	[$-4268$, 7505]	[$-4848$, 2500]

Tables 4 and 5 show that the optimization model can further reduce the weight of the qubits and the range of the coupler strength involved in the problem model, which can advance the large-scale integers in the D-Wave machine.

**Table 5:** The parameter values of our method for integer factorization.

Integers	$p\times q$	h ranges	J ranges
143	$11\times 13$	[$-85$, 86.5]	[$-109$, 66]
59989	$251\times 239$	[$-928$, 1867]	[$-1168$, 701]
376289	$659\times 571$	[$-2886$, 5039]	[$-2103$, 2048]
1005973	$997\times 1009$	[$-3391$, 4860]	[$-2048$, 2048]
1028171	$1009\times 1019$	[$-3005$, 5032]	[$-2078$, 2048]

Table 6 shows a comparison of the different algorithms when factoring the integer 7778 = $31\times 251$.

**Table 6:** Comparison of different algorithms when factoring the integer $31\times 251$ = 7781.

Models	Local field coefficient $h$	Coupler strength $J$	qubits
Ref. ^34^	$1{0}^{6}$	$1{0}^{6}$	419
Ref. ^32^	1256	872	27
Our method	758	554	27

Note: The values of the local field coefficient $h$ and coupler strength $J$ are the absolute values of the parameter ranges. Table 6 takes the maximum integer 7718 that was factored by Warren, R.H.^34^ as an example and compares the coefficients of Ising model and qubits. In the actual quantum annealing experiment, the excessive coupling strength between the qubits reduces the possibility of reaching the ground state, and finally reduces the success rate of the integer factorization. It can be seen from Table 6 that the proposed method achieves the lowest local field coefficient $h$ and coupling coefficient $J$, reduces the ranges of the coefficients of Ising model, and uses far fewer qubits than Warren, R.H.^34^. The reduction of the parameter value ranges can reduce the demand for qubit coupling strength, make the physical qubit flip unified, effectively increase the possibility of quantum annealing reaching the global optimal, and improve the success rate of integer factorization. In the case of insufficient precision and the immature development of existing quantum devices, the proposed method can effectively reduce the hardware requirements and improve the success rate of deciphering RSA via quantum annealing. In addition, our method successfully factors all integers within 10000, whereas Warren, R.H.^34^ traversed and factored all integers within 1000.

## Discussion

The integer factorization method based on the NMR platform uses the special properties of integers, and the method is not universal. The quantum annealing method based on a D-Wave quantum computer for integer factorization is limited by the hardware connection limitations of the D-Wave quantum computer, which are not enough to apply the method to larger integers.

This paper shows the optimistic potential of the quantum annealing algorithm for deciphering the RSA cryptosystem. A D-Wave using quantum annealing provides a new (second) way, which is a completely different way from Shor’s algorithm. The latest IBM Q System One${}^{TM}$ can theoretically factor up to 10-bit integers using Shor’s algorithm, whereas our simulations showed the huge advantages of factoring 20-bit integers (1028171) using the quantum computing software environment provided by D-Wave. Our results are superior to the results obtained by any other quantum algorithm. Compared with ref. ^32^, the local field coefficient $h$ and coupling term coefficient $J$ of Ising model are optimized to reduce the range of the model parameters by more than 33% and 26%, respectively, which reduces the coupling strength between qubits, further improves the stability of qubit chains and further improves the upper bound of integer factorization. With the slow progress of general-purpose quantum computers and the limitation of D-Wave quantum computer’s topological connections, the stability of Ising model can be improved by reducing the local field coefficient $h$ and coupling coefficient $J$ of Ising model, which can effectively improve the upper bounds of the decomposed integers.

From the perspective of practical code-cracking and generalization, we proposed a new general quantum spin model, which is a novel and further scalable way to conduct prime factorization with few qubits and QA. Lockheed Martin’s Warren, R.H.^34^ traversed and factored all integers within 1000. Our method successfully factors all integers within 10000 and has obtained the best index (20-bit integers (1028171)) of quantum computing for factoring integers. The result exceeded the work of Shuxian Jiang *et al*. (factor up to 376289)^30^ and Warren, R.H.^34^ (factor up to 7781).

At present, the fastest classical integer factorization algorithm is the number field sieve method. Its complexity is $O(exp(c{(logN)}^{\frac{1}{3}}){(loglogN)}^{\frac{2}{3}})$ and its complexity is exponential. In theory, Shor’s algorithm requires 2n qubits to factor n-bit integers, where n is the number of binary digits of the integer^13^. The complexity of our method is less than $O(lo{g}^{2}(N))$, where $N$ is the number to be factored. In terms of theoretical complexity, the complexity of Shor’s algorithm is better than the algorithm proposed in this paper. In terms of factoring the maximum integer index, due to the slow development of general quantum devices, Shor’s algorithm currently factor up to integer 85, and the maximum number that can be factored by the integer factorization method based on quantum annealing of our method is integer 1028171. To achieve the factorization of the integer 1028171, Shor’s algorithm requires more than 40 universal qubits, and the number of qubits and the precision of the quantum bits are far beyond the current hardware level. Therefore, through the analysis of the factored maximum integer index, the integer factorization method based on quantum annealing has more realistic attack power than Shor’s algorithm, which is expected to result in more advantages when using the real D-Wave quantum computing platform.

The current state of post quantum cryptography research exclusively referred to the potential threatens of Shor’s algorithm. From the above analysis, it can be seen that quantum annealing (the core principle of the D-Wave quantum computer) for prime factorization may be closer to cracking practical RSA codes than Shor’s algorithm. Furthermore, the experts of the post quantum cryptography international standard organization (in the 6th ETSI/IQC Quantum Safe Workshop) expressed great interest in our method. They analyzed the reason for neglecting the attacks from the D-Wave machine in post quantum cryptography research since the D-Wave computers, which have been purchased by Lockheed Martin, Google, etc., have been initially used for image processing, machine learning, combinatorial optimization, software verification, etc. Thus, post quantum cryptography research should further consider the potential of the D-Wave quantum computer for deciphering the RSA cryptosystem in future.

The structure of large integers will have an impact on the complexity of the model. Future research work will further study the effects of the structure of large integers on the model and the scalability of the integer factorization when using a D-Wave quantum computer to achieve larger-scale integer factorization.

## Supplementary information

Supplementary Information.

## Data Availability

All other data used in this study are available from the corresponding authors upon reasonable request.
