# Supplementary material for: Prime factorization algorithm based on parameter optimization of Ising model
Source: Sci Rep. 2020 Apr 28;10:7106. doi: 10.1038/s41598-020-62802-5 (PMC7188840; doi:10.1038/s41598-020-62802-5)
Supplement: Supplementary file 1 — Supplementary Information. [file 41598_2020_62802_MOESM1_ESM.pdf]

Supplementary material for "Prime factorization algorithm based on parameter optimization of Ising model"

Baonan Wang[1,2], Feng Hu[1,2], Haonan Yao[1,2], and Chao Wang[1,2,3,*]

[1]Key laboratory of Specialty Fiber Optics and Optical Access Networks, Joint International Research Laboratory of Specialty Fiber Optics and Advanced Communication, Shanghai Institute for Advanced Communication and Data Science, Shanghai University, Shanghai, 200444, China
[2]State Key Laboratory of Cryptology, P. O. Box 5159, Beijing, 100878, China
[3]Center for Quantum Computing, Peng Cheng Laboratory, Shenzhen 518000, China
[*]corresponding.wangchao@shu.edu.cn

Factoring $N = 1028171 = 1009 \times 1019$

Table S1 shows the factorization of $N = 1028171 = 1009 \times 1019$.

The first column of the multiplication table is,

$$p_1 + q_1 = 1, \tag{1}$$

we can obtain $p_1 = 1 - q_1$, $p_1q_1 = 0$.

The second column of the multiplication table is,

$$p_2 + p_1 + q_1 + q_2 = 0, \tag{2}$$

we can get $p_2 = q_2$.

The objective function of $N = 1028171 = 1009 \times 1019$ can be given as (Note that the objective function does not include a constant term), $f = 381c_1 + 1276c_2 + 113c_3 + 356c_4 + 1232c_5 - 75c_6 - 20c_7 + 480c_8 + 3061c_9 + 149c_{10} + 428c_{11} + 1376c_{12} + 151c_{13} + 432c_{14} - 36p_3 - 32p_4 - 14p_5 - 24p_6 - 32p_7 + 8p_8 + 108q_2 + 28q_3 - 35q_4 - 14q_5 - 8q_6 - 15q_7 + 48q_8 + 1028c_1c_2 - 16c_1c_3 - 32c_1c_4 - 32c_2c_3 - 64c_1c_5 - 64c_2c_4 - 128c_2c_5 + 260c_3c_4 + 520c_3c_5 - 16c_3c_6 + 1040c_4c_5 - 32c_3c_7 - 32c_4c_6 - 64c_3c_8 - 64c_4c_7 - 64c_5c_6 - 128c_3c_9 - 128c_4c_8 - 128c_5c_7 - 256c_4c_9 - 256c_5c_8 + 260c_6c_7 - 512c_5c_9 + 520c_6c_8 +$

Table S1. Multiplication table for $N = 1028171 = 1009 \times 1019$.

		2^{18}	2^{17}	2^{16}	2^{15}	2^{14}	2^{13}	2^{12}	2^{11}	2^{10}	2^9	2^8	2^7	2^6	2^5	2^4	2^3	2^2	2^1	2^0
p											1	p_8	p_7	p_6	p_5	p_4	p_3	p_2	p_1	1
q											1	q_8	q_7	q_6	q_5	q_4	q_3	q_2	q_1	1
											1	p_8	p_7	p_6	p_5	p_4	p_3	p_2	p_1	1
										q_1	p_8q_1	p_7q_1	p_6q_1	p_5q_1	p_4q_1	p_3q_1	p_2q_1	p_1q_1	q_1	
									q_2	p_8q_2	p_7q_2	p_6q_2	p_5q_2	p_4q_2	p_3q_2	p_2q_2	p_1q_2	q_2		
								q_3	p_8q_3	p_7q_3	p_6q_3	p_5q_3	p_4q_3	p_3q_3	p_2q_3	p_1q_3	q_3			
							q_4	p_8q_4	p_7q_4	p_6q_4	p_5q_4	p_4q_4	p_3q_4	p_2q_4	p_1q_4	q_4				
						q_5	p_8q_5	p_7q_5	p_6q_5	p_5q_5	p_4q_5	p_3q_5	p_2q_5	p_1q_5	q_5					
					q_6	p_8q_6	p_7q_6	p_6q_6	p_5q_6	p_4q_6	p_3q_6	p_2q_6	p_1q_6	q_6						
				q_7	p_8q_7	p_7q_7	p_6q_7	p_5q_7	p_4q_7	p_3q_7	p_2q_7	p_1q_7	q_7							
			q_8	p_8q_8	p_7q_8	p_6q_8	p_5q_8	p_4q_8	p_3q_8	p_2q_8	p_1q_8	q_8								
		1	p_8	p_7	p_6	p_5	p_4	p_3	p_2	p_1	1									
carries		c_{14}	c_{13}			c_9	c_8	c_7	c_6	c_5	c_4	c_3		c_2	c_1					
				c_{12}	c_{11}	c_{10}														
$p \times q$	1	1	1	1	1	0	1	1	0	0	0	0	0	1	0	0	1	0	1	1

$1024c_6c_9+1040c_7c_8-16c_6c_{10}+2048c_7c_9-32c_6c_{11}-32c_7c_{10}+4096c_8c_9-64c_6c_{12}-64c_7c_{11}-64c_8c_{10}-128c_7c_{12}-128c_8c_{11}+$
$2c_9c_{10}-256c_8c_{12}+4c_9c_{11}+8c_9c_{12}+260c_{10}c_{11}-16c_9c_{13}+520c_{10}c_{12}-32c_9c_{14}-16c_{10}c_{13}+1040c_{11}c_{12}-32c_{10}c_{14}-32c_{11}c_{13}-$
$64c_{11}c_{14}-64c_{12}c_{13}-128c_{12}c_{14}+260c_{13}c_{14}-128c_1p_3-256c_1p_4-256c_2p_3+2c_1p_5-512c_2p_4+4c_1p_6+4c_2p_5+8c_1p_7+$
$8c_2p_6-16c_3p_5+16c_2p_7-32c_3p_6-32c_4p_5+4c_6p_3-64c_3p_7-64c_4p_6-64c_5p_5+8c_6p_4+8c_7p_3+2c_3p_8-128c_4p_7-$
$128c_5p_6+16c_7p_4+16c_8p_3+4c_4p_8-256c_5p_7+32c_8p_4+8c_5p_8-32c_{10}p_3-16c_6p_8+2c_9p_5-64c_{10}p_4-64c_{11}p_3-32c_7p_8+$
$4c_9p_6+2c_{10}p_5-128c_{11}p_4-128c_{12}p_3-64c_8p_8+8c_9p_7+4c_{10}p_6+4c_{11}p_5-256c_{12}p_4-128c_9p_8+8c_{10}p_7+8c_{11}p_6+8c_{12}p_5+$
$16c_{11}p_7+16c_{12}p_6-16c_{13}p_5+32c_{12}p_7-32c_{13}p_6-32c_{14}p_5-64c_{13}p_7-64c_{14}p_6+2c_{13}p_8-128c_{14}p_7+4c_{14}p_8-512c_1q_2-$
$384c_1q_3-1024c_2q_2-254c_1q_4-768c_2q_3+6c_1q_5-508c_2q_4+12c_1q_6+12c_2q_5-16c_3q_4+8c_1q_7+24c_2q_6-48c_3q_5-32c_4q_4+$
$4c_6q_2+16c_2q_7-96c_3q_6-96c_4q_5-64c_5q_4+4c_6q_3+8c_7q_2-62c_3q_7-192c_4q_6-192c_5q_5+8c_6q_4+8c_7q_3+16c_8q_2+6c_3q_8-$
$124c_4q_7-384c_5q_6+16c_7q_4+16c_8q_3+12c_4q_8-248c_5q_7+32c_8q_4-32c_{10}q_2+24c_5q_8-16c_6q_7-32c_{10}q_3-64c_{11}q_2-$
$48c_6q_8-32c_7q_7+2c_9q_5-64c_{10}q_4-64c_{11}q_3-128c_{12}q_2-96c_7q_8-64c_8q_7+4c_9q_6+2c_{10}q_5-128c_{11}q_4-128c_{12}q_3-192c_8q_8-$
$120c_9q_7+4c_{10}q_6+4c_{11}q_5-256c_{12}q_4-384c_9q_8+8c_{10}q_7+8c_{11}q_6+8c_{12}q_5+16c_{11}q_7+16c_{12}q_6-16c_{13}q_5+32c_{12}q_7-$
$32c_{13}q_6-32c_{14}q_5-64c_{13}q_7-64c_{14}q_6+2c_{13}q_8-128c_{14}q_7+4c_{14}q_8+80p_3p_4+8p_5p_6+16p_5p_7+32p_6p_7+64p_3q_1+133p_3q_2-$
$3p_4q_1+100p_3q_3+268p_4q_2+88p_3q_4+208p_4q_3+8p_5q_2+16p_6q_1+17p_3q_5+177p_4q_4+17p_5q_3+17p_6q_2+17p_7q_1+36p_3q_6+$
$36p_4q_5+38p_5q_4+36p_6q_3+36p_7q_2+40p_8q_1+88p_3q_7+80p_4q_6+88p_5q_5+84p_6q_4+80p_7q_3+88p_8q_2-7p_3q_8-3p_4q_7+$
$5p_5q_6+5p_6q_5+5p_7q_4-7p_8q_3-4p_4q_8-4p_5q_7+12p_6q_6+12p_7q_5-4p_8q_4-32p_5q_8+32p_7q_6-32p_8q_5-7p_6q_8+69p_7q_7-$
$7p_8q_6-4p_7q_8-2p_8q_7-24p_8q_8-64q_1q_3+3q_1q_4+389q_2q_3+272q_2q_4-16q_1q_6+24q_2q_5+208q_3q_4-17q_1q_7+17q_2q_6-$
$40q_1q_8+40q_2q_7+6q_4q_5+104q_2q_8+12q_4q_6+8q_4q_7+40q_5q_6+32q_5q_7+64q_6q_7+6q_7q_8-256c_1p_3q_1+2c_1p_3q_2+2c_1p_4q_1-$
$512c_2p_3q_1+4c_1p_3q_3+4c_1p_4q_2+4c_1p_5q_1+4c_2p_3q_2+4c_2p_4q_1+8c_1p_3q_4+8c_1p_4q_3+8c_1p_5q_2+8c_1p_6q_1+8c_2p_3q_3+8c_2p_4q_2+$
$8c_2p_5q_1-16c_3p_3q_2-16c_3p_4q_1+16c_2p_3q_4+16c_2p_4q_3+16c_2p_5q_2+16c_2p_6q_1-32c_3p_3q_3-32c_3p_4q_2-32c_3p_5q_1-32c_4p_3q_2-$
$32c_4p_4q_1-64c_3p_3q_4-64c_3p_4q_3-64c_3p_5q_2-64c_3p_6q_1-64c_4p_3q_3-64c_4p_4q_2-64c_4p_5q_1-64c_5p_3q_2-64c_5p_4q_1+2c_3p_3q_5+$
$2c_3p_4q_4+2c_3p_5q_3+2c_3p_6q_2+2c_3p_7q_1-128c_4p_3q_4-128c_4p_4q_3-128c_4p_5q_2-128c_4p_6q_1-128c_5p_3q_3-128c_5p_4q_2-$
$128c_5p_5q_1+4c_3p_3q_6+4c_3p_4q_5+4c_3p_5q_4+4c_3p_6q_3+4c_3p_7q_2+4c_3p_8q_1+4c_4p_3q_5+4c_4p_4q_4+4c_4p_5q_3+4c_4p_6q_2+4c_4p_7q_1-$
$256c_5p_3q_4-256c_5p_4q_3-256c_5p_5q_2-256c_5p_6q_1+8c_3p_3q_7+8c_3p_4q_6+8c_3p_5q_5+8c_3p_6q_4+8c_3p_7q_3+8c_3p_8q_2+8c_4p_3q_6+$
$8c_4p_4q_5+8c_4p_5q_4+8c_4p_6q_3+8c_4p_7q_2+8c_4p_8q_1+8c_5p_3q_5+8c_5p_4q_4+8c_5p_5q_3+8c_5p_6q_2+8c_5p_7q_1+16c_4p_3q_7+16c_4p_4q_6+$
$16c_4p_5q_5+16c_4p_6q_4+16c_4p_7q_3+16c_4p_8q_2+16c_5p_3q_6+16c_5p_4q_5+16c_5p_5q_4+16c_5p_6q_3+16c_5p_7q_2+16c_5p_8q_1-16c_6p_3q_5-$
$16c_6p_4q_4-16c_6p_5q_3-16c_6p_6q_2-16c_6p_7q_1+32c_5p_3q_7+32c_5p_4q_6+32c_5p_5q_5+32c_5p_6q_4+32c_5p_7q_3+32c_5p_8q_2-32c_6p_3q_6-$
$32c_6p_4q_5-32c_6p_5q_4-32c_6p_6q_3-32c_6p_7q_2-32c_6p_8q_1-32c_7p_3q_5-32c_7p_4q_4-32c_7p_5q_3-32c_7p_6q_2-32c_7p_7q_1-64c_6p_3q_7-$
$64c_6p_4q_6-64c_6p_5q_5-64c_6p_6q_4-64c_6p_7q_3-64c_6p_8q_2-64c_7p_3q_6-64c_7p_4q_5-64c_7p_5q_4-64c_7p_6q_3-64c_7p_7q_2-64c_7p_8q_1-$
$64c_8p_3q_5-64c_8p_4q_4-64c_8p_5q_3-64c_8p_6q_2-64c_8p_7q_1+2c_6p_3q_8+2c_6p_4q_7+2c_6p_5q_6+2c_6p_6q_5+2c_6p_7q_4+2c_6p_8q_3-$
$128c_7p_3q_7-128c_7p_4q_6-128c_7p_5q_5-128c_7p_6q_4-128c_7p_7q_3-128c_7p_8q_2-128c_8p_3q_6-128c_8p_4q_5-128c_8p_5q_4-128c_8p_6q_3-$
$128c_8p_7q_2-128c_8p_8q_1-128c_9p_3q_5-128c_9p_4q_4-128c_9p_5q_3-128c_9p_6q_2-128c_9p_7q_1+4c_6p_4q_8+4c_6p_5q_7+4c_6p_6q_6+$
$4c_6p_7q_5+4c_6p_8q_4+4c_7p_3q_8+4c_7p_4q_7+4c_7p_5q_6+4c_7p_6q_5+4c_7p_7q_4+4c_7p_8q_3-256c_8p_3q_7-256c_8p_4q_6-256c_8p_5q_5-$
$256c_8p_6q_4-256c_8p_7q_3-256c_8p_8q_2-256c_9p_3q_6-256c_9p_4q_5-256c_9p_5q_4-256c_9p_6q_3-256c_9p_7q_2-256c_9p_8q_1+8c_6p_5q_8+$
$8c_6p_6q_7+8c_6p_7q_6+8c_6p_8q_5+8c_7p_4q_8+8c_7p_5q_7+8c_7p_6q_6+8c_7p_7q_5+8c_7p_8q_4+8c_8p_3q_8+8c_8p_4q_7+8c_8p_5q_6+8c_8p_6q_5+$
$8c_8p_7q_4+8c_8p_8q_3-512c_9p_3q_7-512c_9p_4q_6-512c_9p_5q_5-512c_9p_6q_4-512c_9p_7q_3-512c_9p_8q_2+16c_7p_5q_8+16c_7p_6q_7+$
$16c_7p_7q_6+16c_7p_8q_5+16c_8p_4q_8+16c_8p_5q_7+16c_8p_6q_6+16c_8p_7q_5+16c_8p_8q_4+32c_8p_5q_8+32c_8p_6q_7+32c_8p_7q_6+32c_8p_8q_5-$
$16c_{10}p_3q_8-16c_{10}p_4q_7-16c_{10}p_5q_6-16c_{10}p_6q_5-16c_{10}p_7q_4-16c_{10}p_8q_3-32c_{10}p_4q_8-32c_{10}p_5q_7-32c_{1}0p_6q_6-32c_{10}p_7q_5-$
$32c_{10}p_8q_4-32c_{11}p_3q_8-32c_{11}p_4q_7-32c_{11}p_5q_6-32c_{1}1p_6q_5-32c_{1}1p_7q_4-32c_{1}1p_8q_3+2c_9p_6q_8+2c_9p_7q_7+2c_9p_8q_6-$
$64c_{10}p_5q_8-64c_{10}p_6q_7-64c_{10}p_7q_6-64c_{10}p_8q_5-64c_{1}1p_4q_8-64c_{1}1p_5q_7-64c_{1}1p_6q_6-64c_{11}p_7q_5-64c_{11}p_8q_4-64c_{1}2p_3q_8-$
$64c_{1}2p_4q_7-64c_{1}2p_5q_6-64c_{1}2p_6q_5-64c_{12}p_7q_4-64c_{12}p_8q_3+4c_9p_7q_8+4c_9p_8q_7+2c_{10}p_6q_8+2c_{10}p_7q_7+2c_{10}p_8q_6-$
$128c_{11}p_5q_8-128c_{11}p_6q_7-128c_{11}p_7q_6-128c_{11}p_8q_5-128c_{12}p_4q_8-128c_{12}p_5q_7-128c_{1}2p_6q_6-128c_{1}2p_7q_5-128c_{1}2p_8q_4+$
$8c_9p_8q_8+4c_{10}p_7q_8+4c_{10}p_8q_7+4c_{11}p_6q_8+4c_{11}p_7q_7+4c_{11}p_8q_6-256c_{1}2p_5q_8-256c_{1}2p_6q_7-256c_{1}2p_7q_6-256c_{1}2p_8q_5+$
$8c_{10}p_8q_8+8c_{11}p_7q_8+8c_{11}p_8q_7+8c_{12}p_6q_8+8c_{12}p_7q_7+8c_{12}p_8q_6+16c_{11}p_8q_8+16c_{12}p_7q_8+16c_{12}p_8q_7-16c_{13}p_6q_8-$
$16c_{13}p_7q_7-16c_{13}p_8q_6+32c_{12}p_8q_8-32c_{13}p_7q_8-32c_{13}p_8q_7-32c_{14}p_6q_8-32c_{14}p_7q_7-32c_{14}p_8q_6-64c_{13}p_8q_8-64c_{1}4p_7q_8-$
$64c_{14}p_8q_7-128c_{1}4p_8q_8+256c_1q_1q_3-2c_1q_1q_4+2c_1q_2q_3+512c_2q_1q_3-4c_1q_1q_5+4c_1q_2q_4-4c_2q_1q_4+4c_2q_2q_3-8c_1q_1q_6+$
$8c_1q_2q_5-8c_2q_1q_5+8c_2q_2q_4+16c_3q_1q_4-16c_3q_2q_3-16c_2q_1q_6+16c_2q_2q_5+32c_3q_1q_5-32c_3q_2q_4+32c_4q_1q_4-32c_4q_2q_3+$
$64c_3q_1q_6-64c_3q_2q_5+64c_4q_1q_5-64c_4q_2q_4+64c_5q_1q_4-64c_5q_2q_3-2c_3q_1q_7+2c_3q_2q_6+128c_4q_1q_6-128c_4q_2q_5+128c_5q_1q_5-$
$128c_5q_2q_4-4c_3q_1q_8+4c_3q_2q_7-4c_4q_1q_7+4c_4q_2q_6+256c_5q_1q_6-256c_5q_2q_5+8c_3q_2q_8-8c_4q_1q_8+8c_4q_2q_7-8c_5q_1q_7+$
$8c_5q_2q_6+16c_4q_2q_8-16c_5q_1q_8+16c_5q_2q_7+16c_6q_1q_7-16c_6q_2q_6+32c_5q_2q_8+32c_6q_1q_8-32c_6q_2q_7+32c_7q_1q_7-32c_7q_2q_6-$
$64c_6q_2q_8+64c_7q_1q_8-64c_7q_2q_7+64c_8q_1q_7-64c_8q_2q_6-128c_7q_2q_8+128c_8q_1q_8-128c_8q_2q_7+128c_9q_1q_7-128c_9q_2q_6-$
$256c_8q_2q_8+256c_9q_1q_8-256c_9q_2q_7-512c_9q_2q_8+128p_3p_4q_1+4p_3p_4q_2+16p_3p_4q_3+10p_3p_5q_2+6p_4p_5q_1+4p_3p_5q_3+$
$4p_3p_6q_2+20p_4p_5q_2+12p_4p_6q_1+4p_3p_4q_5+8p_3p_5q_4+8p_3p_6q_3+8p_3p_7q_2+8p_4p_5q_3+8p_4p_6q_2+8p_4p_7q_1+32p_5p_6q_1+$
$16p_3p_4q_6+8p_3p_5q_5+16p_3p_6q_4+16p_3p_7q_3+4p_4p_5q_4+16p_4p_6q_3+16p_4p_7q_2+16p_5p_6q_2+16p_5p_7q_1+4p_3p_4q_7+4p_3p_5q_6+$
$4p_3p_6q_5+36p_3p_7q_4+4p_3p_8q_3+16p_4p_5q_5+8p_4p_6q_4+32p_4p_7q_3+4p_5p_6q_3+32p_5p_7q_2+32p_6p_7q_1+20p_3p_4q_8+8p_3p_5q_7+$

$8p_3p_6q_6+8p_3p_7q_5+8p_3p_8q_4+8p_4p_5q_6+8p_4p_6q_5+8p_4p_7q_4+8p_4p_8q_3+16p_5p_6q_4+8p_5p_7q_3+4p_6p_7q_2+24p_3p_5q_8+$
$16p_3p_6q_7+16p_3p_7q_6+18p_3p_8q_5+20p_4p_5q_7+16p_4p_6q_6+16p_4p_7q_5+18p_4p_8q_4+2p_5p_8q_3+16p_6p_7q_3+10p_6p_8q_2+$
$6p_7p_8q_1+4p_3p_8q_6+48p_4p_5q_8+40p_4p_6q_7+32p_4p_7q_6+36p_4p_8q_5+4p_5p_6q_6+4p_5p_8q_4+4p_6p_8q_3+20p_7p_8q_2+8p_3p_8q_7+$
$8p_4p_8q_6+16p_5p_6q_7+8p_5p_7q_6+8p_5p_8q_5+4p_6p_7q_5+8p_6p_8q_4+8p_7p_8q_3+2p_5p_6q_8+2p_5p_7q_7+2p_5p_8q_6+16p_6p_7q_6+$
$8p_6p_8q_5+4p_7p_8q_4+4p_5p_7q_8+4p_5p_8q_7+4p_6p_7q_7+4p_6p_8q_6+16p_7p_8q_5+8p_5p_8q_8+20p_6p_7q_8+8p_6p_8q_7+8p_7p_8q_6+$
$24p_6p_8q_8+20p_7p_8q_7+48p_7p_8q_8+256p_3q_1q_2+4p_4q_1q_2+120p_3q_1q_4+10p_3q_2q_3-120p_4q_1q_3+16p_5q_1q_2+30p_3q_2q_4+$
$28p_4q_2q_3+14p_3q_2q_5+20p_3q_3q_4+2p_4q_1q_5+12p_4q_2q_4-2p_5q_1q_4+10p_5q_2q_3+4p_7q_1q_2-8p_3q_1q_7+16p_3q_2q_6+12p_3q_3q_5+$
$4p_4q_1q_6+28p_4q_2q_5+8p_4q_3q_4+28p_5q_2q_4-4p_6q_1q_4+8p_6q_2q_3+8p_7q_1q_3+16p_8q_1q_2+24p_3q_2q_7+24p_3q_3q_6+24p_3q_4q_5+$
$8p_4q_1q_7+32p_4q_2q_6+24p_4q_3q_5+64p_5q_2q_5+4p_5q_3q_4+16p_6q_2q_4-8p_7q_1q_4+24p_7q_2q_3+4p_3q_2q_8+16p_3q_3q_7+48p_3q_4q_6+$
$20p_4q_2q_7+48p_4q_3q_6+4p_4q_4q_5+16p_5q_1q_7+52p_5q_2q_6+8p_5q_3q_5+20p_6q_2q_5+16p_6q_3q_4-16p_7q_1q_5+20p_7q_2q_4+4p_8q_2q_3+$
$4p_3q_3q_8+32p_3q_4q_7+4p_3q_5q_6+8p_4q_2q_8+36p_4q_3q_7+8p_4q_4q_6+40p_5q_2q_7+4p_5q_3q_6+16p_5q_4q_5+32p_6q_1q_7+10p_6q_2q_6+$
$4p_6q_3q_5-32p_7q_1q_6+40p_7q_2q_5+4p_7q_3q_4+8p_8q_2q_4+8p_3q_4q_8+10p_3q_5q_7+8p_4q_3q_8+10p_4q_4q_7+16p_4q_5q_6+16p_5q_2q_8+$
$10p_5q_3q_7+8p_5q_4q_6+22p_6q_2q_7+8p_6q_3q_6+8p_6q_4q_5+20p_7q_2q_6+8p_7q_3q_5+16p_8q_2q_5+20p_8q_3q_4+6p_3q_5q_8+20p_3q_6q_7+$
$22p_4q_4q_8+4p_4q_5q_7+22p_5q_3q_8+20p_5q_4q_7+14p_6q_2q_8+20p_6q_3q_7+16p_6q_4q_6+2p_7q_1q_8+12p_7q_2q_7+16p_7q_3q_6+20p_7q_4q_5-$
$2p_8q_1q_7+10p_8q_2q_6+24p_8q_3q_5+12p_3q_6q_8+12p_4q_5q_8+8p_4q_6q_7+44p_5q_4q_8+8p_5q_5q_7+12p_6q_3q_8+40p_6q_4q_7+4p_6q_5q_6+$
$28p_7q_2q_8+8p_7q_3q_7+40p_7q_4q_6+28p_8q_2q_7+48p_8q_4q_5+24p_3q_7q_8+24p_4q_6q_8+24p_5q_5q_8+4p_5q_6q_7+24p_6q_4q_8+8p_6q_5q_7+$
$24p_7q_3q_8+16p_7q_5q_6+64p_8q_2q_8+4p_4q_7q_8+8p_5q_6q_8+2p_6q_5q_8+16p_6q_6q_7+2p_7q_5q_7+2p_8q_5q_6+16p_5q_7q_8+4p_6q_6q_8+$
$4p_7q_5q_8+4p_7q_6q_7+4p_8q_5q_7+8p_6q_7q_8+8p_7q_6q_8+8p_8q_5q_8+20p_8q_6q_7+20p_7q_7q_8+24p_8q_6q_8+48p_8q_7q_8-256q_1q_2q_3-$
$4q_1q_2q_4-16q_1q_2q_5-128q_1q_3q_4+6q_2q_3q_4-4q_1q_2q_7-6q_1q_4q_5+14q_2q_3q_5-16q_1q_2q_8-12q_1q_4q_6+12q_2q_3q_6+36q_2q_4q_5-$
$8q_1q_4q_7-32q_1q_5q_6+8q_2q_3q_7+24q_2q_4q_6-16q_1q_5q_7+16q_2q_4q_7+48q_2q_5q_6-32q_1q_6q_7+32q_2q_5q_7+6q_2q_6q_7-6q_1q_7q_8+$
$14q_2q_6q_8+36q_2q_7q_8+2p_3p_4q_1q_2+4p_3p_4q_1q_3+4p_3p_5q_1q_2+8p_3p_4q_1q_4+16p_3p_4q_2q_3+8p_3p_5q_1q_3+8p_3p_6q_1q_2+16p_4p_5q_1q_2+$
$16p_3p_4q_2q_4+16p_3p_5q_1q_4+16p_3p_5q_2q_3+16p_3p_6q_1q_3+16p_4p_5q_1q_3+16p_4p_6q_1q_2+32p_3p_4q_3q_4+32p_3p_5q_2q_4+32p_3p_6q_1q_4+$
$32p_4p_5q_2q_3+32p_4p_6q_1q_3+32p_5p_6q_1q_2+2p_3p_4q_4q_5+2p_3p_5q_3q_5+2p_3p_6q_2q_5+2p_3p_7q_1q_5+2p_4p_5q_3q_4+2p_4p_6q_2q_4+$
$2p_4p_7q_1q_4+2p_5p_6q_2q_3+2p_5p_7q_1q_3+2p_6p_7q_1q_2+4p_3p_4q_4q_6+4p_3p_5q_3q_6+4p_3p_5q_4q_5+4p_3p_6q_2q_6+4p_3p_6q_3q_5+4p_3p_7q_1q_6+$
$4p_3p_7q_2q_5+4p_3p_8q_1q_5+4p_4p_5q_3q_5+4p_4p_6q_2q_5+4p_4p_6q_3q_4+4p_4p_7q_1q_5+4p_4p_7q_2q_4+4p_4p_8q_1q_4+4p_5p_6q_2q_4+4p_5p_7q_1q_4+$
$4p_5p_7q_2q_3+4p_5p_8q_1q_3+4p_6p_7q_1q_3+4p_6p_8q_1q_2+8p_3p_4q_4q_7+16p_3p_4q_5q_6+8p_3p_5q_3q_7+8p_3p_5q_4q_6+8p_3p_6q_2q_7+8p_3p_6q_3q_6+$
$8p_3p_6q_4q_5+8p_3p_7q_1q_7+8p_3p_7q_2q_6+8p_3p_7q_3q_5+8p_3p_8q_1q_6+8p_3p_8q_2q_5+8p_4p_5q_3q_6+16p_4p_5q_4q_5+8p_4p_6q_2q_6+8p_4p_6q_3q_5+$
$8p_4p_7q_1q_6+8p_4p_7q_2q_5+8p_4p_7q_3q_4+8p_4p_8q_1q_5+8p_4p_8q_2q_4+8p_5p_6q_2q_5+16p_5p_6q_3q_4+8p_5p_7q_1q_5+8p_5p_7q_2q_4+8p_5p_8q_1q_4+$
$8p_5p_8q_2q_3+8p_6p_7q_1q_4+16p_6p_7q_2q_3+8p_6p_8q_1q_3+16p_7p_8q_1q_2+16p_3p_4q_5q_7+16p_3p_5q_4q_7+16p_3p_5q_5q_6+16p_3p_6q_3q_7+$
$16p_3p_6q_4q_6+16p_3p_7q_2q_7+16p_3p_7q_3q_6+16p_3p_8q_1q_7+16p_3p_8q_2q_6+16p_4p_5q_4q_6+16p_4p_6q_3q_6+16p_4p_6q_4q_5+16p_4p_7q_2q_6+$
$16p_4p_7q_3q_5+16p_4p_8q_1q_6+16p_4p_8q_2q_5+16p_5p_6q_3q_5+16p_5p_7q_2q_5+16p_5p_7q_3q_4+16p_5p_8q_1q_5+16p_5p_8q_2q_4+16p_6p_7q_2q_4+$
$16p_6p_8q_1q_4+16p_6p_8q_2q_3+16p_7p_8q_1q_3+32p_3p_4q_6q_7+32p_3p_5q_5q_7+32p_3p_6q_4q_7+32p_3p_7q_3q_7+32p_3p_8q_2q_7+32p_4p_5q_5q_6+$
$32p_4p_6q_4q_6+32p_4p_7q_3q_6+32p_4p_8q_2q_6+32p_5p_6q_4q_5+32p_5p_7q_3q_5+32p_5p_8q_2q_5+32p_6p_7q_3q_4+32p_6p_8q_2q_4+32p_7p_8q_2q_3+$
$2p_3p_4q_7q_8+2p_3p_5q_6q_8+2p_3p_6q_5q_8+2p_3p_7q_4q_8+2p_3p_8q_3q_8+2p_4p_5q_6q_7+2p_4p_6q_5q_7+2p_4p_7q_4q_7+2p_4p_8q_3q_7+2p_5p_6q_5q_6+$
$2p_5p_7q_4q_6+2p_5p_8q_3q_6+2p_6p_7q_4q_5+2p_6p_8q_3q_5+2p_7p_8q_3q_4+4p_3p_5q_7q_8+4p_3p_6q_6q_8+4p_3p_7q_5q_8+4p_3p_8q_4q_8+4p_4p_5q_6q_8+$
$4p_4p_6q_5q_8+4p_4p_6q_6q_7+4p_4p_7q_4q_8+4p_4p_7q_5q_7+4p_4p_8q_3q_8+4p_4p_8q_4q_7+4p_5p_6q_5q_7+4p_5p_7q_4q_7+4p_5p_7q_5q_6+4p_5p_8q_3q_7+$
$4p_5p_8q_4q_6+4p_6p_7q_4q_6+4p_6p_8q_3q_6+4p_6p_8q_4q_5+4p_7p_8q_3q_5+8p_3p_6q_7q_8+8p_3p_7q_6q_8+8p_3p_8q_5q_8+16p_4p_5q_7q_8+8p_4p_6q_6q_8+$
$8p_4p_7q_5q_8+8p_4p_7q_6q_7+8p_4p_8q_4q_8+8p_4p_8q_5q_7+8p_5p_6q_5q_8+16p_5p_6q_6q_7+8p_5p_7q_4q_8+8p_5p_7q_5q_7+8p_5p_8q_3q_8+8p_5p_8q_4q_7+$
$8p_5p_8q_5q_6+8p_6p_7q_4q_7+16p_6p_7q_5q_6+8p_6p_8q_3q_7+8p_6p_8q_4q_6+8p_7p_8q_3q_6+16p_7p_8q_4q_5+16p_4p_6q_7q_8+16p_4p_7q_6q_8+$
$16p_4p_8q_5q_8+16p_5p_6q_6q_8+16p_5p_7q_5q_8+16p_5p_7q_6q_7+16p_5p_8q_4q_8+16p_5p_8q_5q_7+16p_6p_7q_5q_7+16p_6p_8q_4q_7+16p_6p_8q_5q_6+$
$16p_7p_8q_4q_6+32p_5p_6q_7q_8+32p_5p_7q_6q_8+32p_5p_8q_5q_8+32p_6p_7q_6q_7+32p_6p_8q_5q_7+32p_7p_8q_5q_6+2p_6p_7q_7q_8+2p_6p_8q_6q_8+$
$2p_7p_8q_6q_7+4p_6p_8q_7q_8+4p_7p_8q_6q_8+16p_7p_8q_7q_8-2p_3q_1q_2q_4+2p_4q_1q_2q_3-4p_3q_1q_2q_5-4p_3q_1q_3q_4+4p_5q_1q_2q_3-8p_3q_1q_2q_6-$
$8p_3q_1q_3q_5+16p_3q_2q_3q_4-8p_4q_1q_3q_4+8p_6q_1q_2q_3-16p_3q_1q_3q_6-16p_3q_1q_4q_5+16p_3q_2q_3q_5-16p_4q_1q_2q_6-16p_4q_1q_3q_5+$
$16p_4q_2q_3q_4+16p_6q_1q_2q_4-32p_3q_1q_4q_6+32p_3q_2q_4q_5-32p_4q_1q_3q_6+32p_4q_2q_3q_5-32p_5q_1q_2q_6+32p_6q_1q_2q_5-2p_3q_1q_5q_7+$
$2p_3q_2q_5q_6-2p_4q_1q_4q_7+2p_4q_2q_4q_6-2p_5q_1q_3q_7+2p_5q_2q_3q_6-2p_6q_1q_2q_7+2p_7q_1q_2q_6-4p_3q_1q_5q_8-4p_3q_1q_6q_7+4p_3q_2q_5q_7-$
$4p_4q_1q_4q_8-4p_4q_1q_5q_7+4p_4q_2q_4q_7+4p_4q_2q_5q_6-4p_5q_1q_3q_8-4p_5q_1q_4q_7+4p_5q_2q_3q_7+4p_5q_2q_4q_6-4p_6q_1q_2q_8-4p_6q_1q_3q_7+$
$4p_6q_2q_3q_6+4p_8q_1q_2q_6-8p_3q_1q_6q_8+8p_3q_2q_5q_8+16p_3q_2q_6q_7-8p_4q_1q_5q_8-8p_4q_1q_6q_7+8p_4q_2q_4q_8+8p_4q_2q_5q_7-8p_5q_1q_4q_8-$
$8p_5q_1q_5q_7+8p_5q_2q_3q_8+8p_5q_2q_4q_7+8p_5q_2q_5q_6-8p_6q_1q_3q_8-8p_6q_1q_4q_7+8p_6q_2q_3q_7+8p_6q_2q_4q_6-8p_7q_1q_3q_7+8p_7q_2q_3q_6-$
$16p_3q_1q_7q_8+16p_3q_2q_6q_8-16p_4q_1q_6q_8+16p_4q_2q_5q_8+16p_4q_2q_6q_7-16p_5q_1q_5q_8+16p_5q_2q_4q_8+16p_5q_2q_5q_7-16p_6q_1q_4q_8+$
$16p_6q_2q_3q_8+16p_6q_2q_4q_7-16p_7q_1q_3q_8+16p_7q_2q_3q_7+32p_3q_2q_7q_8+32p_4q_2q_6q_8+32p_5q_2q_5q_8+32p_6q_2q_4q_8+32p_7q_2q_3q_8-$
$2q_1q_2q_3q_4-4q_1q_2q_3q_5-8q_1q_2q_3q_6-16q_1q_2q_4q_5-16q_1q_2q_4q_6-32q_1q_2q_5q_6-2q_1q_2q_6q_7-4q_1q_2q_6q_8-16q_1q_2q_7q_8$
